# Conceptualizing acts and behaviours that comprise intimate partner violence: a concept map

**DOI:** 10.1111/hex.12291

**Published:** 2014-11-06

**Authors:** Patricia O'Campo, Janet Smylie, Anita Minh, Mairi Omand, Ajitha Cyriac

**Affiliations:** ^1^ Centre for Research on Inner City Health The Keenan Research Centre in the Li Ka Shing Knowledge Institute of St. Michael's Hospital Toronto ON Canada; ^2^ Dalla Lana School of Public Health University of Toronto Toronto ON Canada

**Keywords:** concept mapping, measurement, partner violence

## Abstract

**Background:**

This study aims to explore the conceptualization of intimate partner violence (IPV) among men and women from diverse subpopulations in Toronto, ON, Canada. Relatively few research efforts have been made to examine differences in conceptualizations of IPV across populations of different race and ethnic backgrounds.

**Methods:**

Using concept mapping methodology, we sampled 67 women and men identified concepts and groups of concepts (domains) that reflected their understandings of the behaviours and attitudes that comprised IPV. We also determined the relative importance of each concept and domain as a contributor to IPV.

**Results:**

‘External and Cultural Influences’, ‘Victim Response to Abuse’ and ‘Social and Emotional Manipulation’ were a few domains that participants rated as moderately or highly important contributors to IPV. These conceptual domains are often left out of commonly used IPV measures.

**Conclusions:**

Our findings have important implications for the conceptualization of IPV and for future IPV measurement and measurement tool development.

## Introduction

Over the past three decades, there has been a long overdue recognition of intimate partner violence (IPV) as a major public health concern, accompanied by a growing formal research agenda on creating measurement and screening tools, and establishing prevalence.^1^, ^2^, ^3^, ^4^, ^5^, ^6^ The World Health Organization (WHO) defines partner violence as ‘behaviour by an intimate partner or ex‐partner that causes physical, sexual or psychological harm, including physical aggression, sexual coercion, psychological abuse and controlling behaviours’.^7^

Estimates on the country‐specific lifetime prevalence of physical and sexual violence against women from partners vary widely across the globe from a low of 11% to a high of 71%.^8^, ^9^ Large‐scale population studies commonly limit measurement of IPV to selected constructs of partner violence and may leave out critical components of IPV (e.g. controlling behaviours).^10^, ^11^, ^12^, ^13^, ^14^ Use of narrow tools has the potential of underestimating IPV prevalence and fails to capture the full impact of partner violence. For example, adverse psychological health consequences of controlling behaviours or battering have been well documented.^15^, ^16^, ^17^

Ideally, our concepts and measures of IPV should also be inclusive of and relevant for the different social and cultural experiences and perspectives represented by the broad racial and ethnic groups of men and women in a population.^18^ Currently, too few instruments capture the full range of behaviours comprising IPV as defined by the WHO. While newer tools are including more constructs such as control,^17^, ^19^ many commonly used measures focus on physical abuse, sexual abuse and psychological abuse. Ethnic minority and socio‐economically disadvantaged populations are particularly affected by narrow measurement tools as mainstream definitions and measures fail to capture the experiences of women from a variety of ethnic backgrounds and women who are experiencing socio‐economic disadvantage.^20^, ^21^, ^22^ Much research on measurement of IPV has been conducted with primarily Caucasian populations from the United States as only within the last 15 years have studies included ethno‐racial populations.^23^ There is an urgent need to expand conceptualizations and measures of IPV to include the experiences of other ethnic groups as each group varies in their cultural norms and perceptions about domestic violence.^2^, ^10^, ^11^, ^20^, ^22^, ^24^, ^25^

Relatively few research efforts have been made to examine differences in conceptualizations of IPV across populations of different racial and ethnic backgrounds with studies of ethnic minorities being under‐represented in the literature.^2^, ^21^, ^22^, ^26^, ^27^, ^28^ In a US national sample differences were reported between Whites, African Americans, Latinas and Asians in their perceptions of what constitutes abuse and domestic violence.^25^ Using vignettes, Klein *et al*.^25^ found that Asian women were the least likely and White women were the most likely to categorize the various interactions such as ‘neighbours having another fight screaming at each other at the top of their lungs’, or ‘at a large family dinner, your cousin is fighting with his wife and shoves her and smacks her across the face’, as domestic violence. In another study that examined British African, Caribbean and White women, the authors report differences in their responses to psychological abuse, in part shaped by cultural factors, as well as, dissimilarities on when to seek or receive ‘formal’ help.^29^ While the gaps identified in the measurement literature include a lack of focus on social, cultural and contextual components of IPV and an absence of perspectives that represent both the victims and perpetrators, there is also an absence of participant driven conceptualizations of the behavioural components of IPV across a diverse population.^19^, ^21^, ^30^

In this study, we use Trochim's method of concept mapping to begin to fill in some gaps in the literature by presenting data on how women and men from diverse ethnic backgrounds conceptualize the behavioural aspects of severe relationship conflict and IPV. This study addresses the following questions: How does a diverse sample of men and women from different levels of socio‐economic position and backgrounds, with respect to ethnicity, and Aboriginal origin, conceptualize relationship violence? Do these conceptualizations differ by personal experience with relationship violence, or by demographic or attitudinal characteristics? Do these constructs differ from commonly employed measures and screening tools for IPV? To address these questions, we used Trochim's methodology of concept mapping, which is a participatory research technique that seeks to clarify the conceptual elements, domains and underlying framework of a phenomenon as experienced by a population of interest.^31^, ^32^ It is a structured process that integrates participants’ input on a single topic and produces an interpretable picture of participants’ ideas, conceptualizations and their internal relatedness.

## Methods

### Recruitment

We undertook this research in a large urban centre characterized by high levels of socio‐economic and ethnic diversity. Recruitment flyers were posted describing opportunities to participate in a group discussion about ‘relationship stress’ in collaborating community organizations where individuals of diverse ethnic and socio‐economically disadvantaged backgrounds seek health and social services (e.g. community organizations and health services serving immigrants and Aboriginal people, community health centres). Trained staff screened interested participants in a private setting to assess eligibility. Our study required participants to be over the age of 18 and to be comfortable speaking, reading and writing in English (as the study activities involved short surveys and manipulation of concepts written in English). At the time of screening, staff explained to participants that the project was about relationship conflict including partner violence. Participants were purposively selected for this study to capture perspectives of individuals from a wide range of ethnic backgrounds, and persons from visible minority groups were oversampled. We undertook two identical waves of recruitment separated by a few months. All in‐person data collection and analysis groups were held at locations in Toronto that were accessible by public transit. All activities were approved by the St. Michael's Hospital Research Ethics Board.

### Data collection

#### Brief survey

Participants completed a brief paper and pencil survey about their demographics, socio‐economic position, ethnic or Aboriginal identity, and experiences of violence and abuse within the relationship, and relationship attitudes. This information was used to characterize our sample and to determine whether ratings differed by participant status with respect to these variables.

#### Brainstorming

Brainstorming was used to generate participant perspectives on behaviours and attitudes that are representative of partner violence. During brainstorming, a focal question was presented to all participants: ‘What are the behaviours or attitudes that would make up the part of the relationship characterized by severe conflict, abuse, excessive control, neglect or even violence?’ Brainstorming took place in group sessions or through a web‐based program specifically designed for concept mapping. Eight gender‐segregated group sessions, led by gender‐matched facilitators and staff, were held (four women's and four men's groups), each approximately 1.5 h in length. Items from all in‐person and online brainstorming sessions were combined, duplicates removed and like items combined. This list of several hundred items had to be reduced to a manageable number for the next stage of sorting and rating. Thus, the remaining items were thematically coded and then pruned within themes such that unique ideas were preserved, to facilitate creation a master list of fewer than 100 items representing what our participants viewed as the universe of all possible notions of partner abuse. One of our thematic codes was ‘not relevant’ to help us weed out items that did not answer the focal question (e.g. ‘superficiality’, ‘poor upbringing’).

#### Sorting and rating items

Sorting items on the master list determines the inter‐relatedness of the ideas identified during the brainstorming activity. All of the participants who took part in the brainstorming were invited to the sorting and rating portion of the study. Participants were given the option of performing the activity in groups supervised by trained facilitators, or online using the aforementioned web‐based program. To complete the sorting activity in person, each participant was given a set of cards with each item written on a single card and told to work independently. Participants were told to sort each individual item into conceptually similar piles. Specifically, participants were asked to group items in a way that ‘made sense to them’ and to label their final groupings. The sorts and the names of the groups for each participant were data entered into the Concept Systems Inc. software by staff for the in‐person sessions or automatically for those individuals participating online.

Rating items determine the relative value of the brainstormed ideas for answering the focal question. Participants were asked to rate each item from the master list on the following question: How important is each item in making up relationship violence, abuse or severe conflict? Responses were recorded as a 5‐point Likert scale (1 – not a central part of the definition, 5 – highly central).

### Analysis

#### Statistical analyses of sorting data

**Figure 1:**
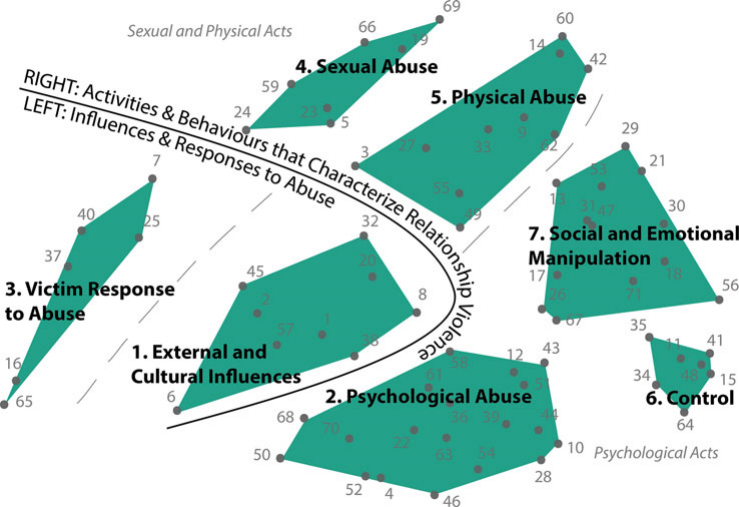
Concept map of 71 elements of relationship stress and violence, based on multidimensional scaling and cluster analysis of all participant data generated from the sorting stage of concept mapping. Major conceptual regions are depicted on the map.

The sorting and rating data underwent two statistical procedures described in detail elsewhere but described briefly here.^33^ First, multidimensional scaling (MDS) was performed on participants’ sorted items to give each item x‐y coordinates for placement on a two‐dimensional plane, followed by hierarchical cluster analysis (HCA) to partition the data into distinct clusters of items. HCA places similar items into non‐overlapping clusters, representing conceptual domains, and the results are displayed as a two‐dimensional cluster map (e.g. see Fig. 1). Statistical analyses were done using the Concept System Core software.^34^ Stress values, a statistic that reflects stability within the clusters and overall map, were generated. Ideal stress values are below 0.36.^31^ Bridging values (the quantification of an item's average conceptual consistency with the items in its vicinity) were generated and can take on a value from 0 to 1. Values closer to 0 denote items that were perceived to be conceptually similar to items within its vicinity, that is an ‘anchoring’ statement; and, a value closer to 1 denotes an item that may have been seen by participants to be conceptually related to many items, both near and far, that is a ‘bridging’ statement. Once the statistical analyses were complete and 2‐dimensional cluster maps generated based on these analyses, we shared this information with our participants to get their input on finalizing the maps.

#### Interpretation by participants

Interpretation of the cluster map involved getting input from participants. Researchers presented to a small group of participants the software‐generated concept map. We held two interpretation sessions (one for men and one for women), designed to allow for participants to shape the configuration of the concept maps. During each of the sessions, participants reviewed the maps for cluster‐naming and within‐cluster coherence of content and suggested individual items that could be moved from one cluster to another, to maintain conceptual clarity. Two modified maps resulted from these interpretation sessions.

A third map, for the entire sample, was then generated based on consolidation of maps from the two interpretation groups. A consolidated map was created by comparing the cluster content and labels across the two modified maps, and combining the maps such that clusters that were similar between them were modified minimally or not at all. For almost all items, the conceptual consistency was very high, that is both interpretation groups placed items in similarly labelled clusters (e.g. an item in the emotional abuse cluster for women was in the psychological abuse or control cluster for men). When there were discrepancies, we sought to maintain within‐cluster conceptual consistency. Using the consolidated map, researchers identified regions of the map that had conceptual similarity. Boundaries and some labels were inserted into the map to indicate the separate the sections.

#### Statistical analyses of rating data

Additionally, participants’ average ratings of clusters and of individual items were analysed, to get a sense of participants’ overall opinions about how central each individual construct or domain appeared to be in defining relationship violence. To compare the extent that ratings differed by subgroups (defined by sociodemographic characteristics, personal experience with IPV and relationship attitudes), Pearson's correlation coefficient (*r*) was calculated from the rating data using Concept Systems software. Pearson's correlation coefficient is a statistical measure of correspondence between two variables, with values ranging from −1.0 to 1.0 where 1.0 indicates a perfect correspondence.

#### Comparison to IPV measures

Finally, we were interested in understanding whether and to what extent constructs of relationship violence that were identified through concept mapping were represented in commonly used measures of partner violence used in research or in screening situations. We identified the main constructs for each instrument and compared it to the constructs from our concept mapping exercise (Table 4). Any domain from the commonly used instruments that were not included in the concept mapping was identified and is listed in the last column of Table 4.

## Results

Sixty‐seven individuals (32 women and 28 men, 7 not stated) participated in brainstorming. Of these, six men and 20 women returned for the sorting and rating activities. An additional 44 individuals (23 men and 22 women) were enlisted, resulting in total of 29 men and 42 women who completed sorting and rating activities. Seventy‐eight per cent of our sample received a post‐secondary degree and age ranged from 30 to 45 years. Twelve per cent of our sample had personal experience with relationship violence. Self‐identified ethnic background was reported as East Asian for 15%, South Asian for 36%, West Indian for 7%, Canadian for 30%, Easter or Western European for 9% of the sample and 18% chose not to report. Aboriginal and African background were reported by one person each.

### Brainstormed items

Table 1 contains a list of 71 items that represent the ideas that were brainstormed by the participants. These 71 unique items were reduced from a much larger list of 876 items generated by participants in the groups and online activity. Figure 1 shows the clustering and location of the 71 items in relation to each other for the overall consolidated group map (see numbers next to the points on the concept map in Fig. 1). Points that are closer together represent items that were sorted together more frequently by participants. Figure 1 (as well as Table 1) also illustrates the items grouped into the consolidated cluster map. Items in Cluster 1 pertain to the factors or circumstances that influence the development or perpetuation of violence within a relationship; these are cultural and/or external to the relationship or dynamic of interaction between the perpetrator or the victim of violence (e.g. *using cultural values to excuse or encourage abuse*,* making sexist and racist remark about the victim, forcing the victim to work for pay, etc*.). Items in Cluster 2 describe a variety of activities or behaviours pertaining to psychological abuse within a relationship (*e.g. turning those in the victim's social network against the victim, maintaining a secret lifestyle, sabotaging the victim's housework, etc*.). Items in Cluster 3 represent an inversion in the directionality of behaviours within a violent relationship, describing the actions that victims take in response to abuse (*e.g. destroying the perpetrator's personal property, screaming and yelling at the perpetrator, ignoring the perpetrator, etc*.). Cluster 4 contains items that describe the actions or behaviours of sexual abuse within a relationship (*e.g. infecting a victim with sexually transmitted infections, punishing the victim for not having sex, keeping the victim from enjoying sex, etc*.). Cluster 5 is composed of a variety of items that describe the actions or behaviours that characterize physical abuse in a relationship (*e.g. beating, slapping, pushing or spitting on the victim; using a weapon to harm the victim; neglecting the victim when they are sick, etc*.). The items in Cluster 6 describe actions of control by the perpetrator over the victim, or controlling behaviours (*e.g. keeping the victim and children separated, restricting the victim's access to education or work, controlling the victim's social contact, etc*.). Finally, Cluster 7 describes the actions or behaviours of social and emotional manipulation (*e.g. encouraging children to take part in violence towards the victim, publicly humiliating the victim, stalking the victim, etc*.).

**Table 1:** Cluster content and cluster average ratings on centrality to the definition of partner violence

No.	Clusters	Rating on the centrality of this cluster to the definition of violence
1	External and Cultural Influences	Moderate
	Perpetrator using their cultural values to excuse abuse or violence	
	Perpetrator publically disclosing details of sex life with victim to show their power	
	Perpetrator punishing victim on issues related to child gender (e.g. blaming women for not having boy child or forcing child gender preference)	
	Perpetrator slanting cultural, religious and moral values to encourage abuse of victim	
	Perpetrator making sexist and racist remarks about victim	
	Perpetrator preventing victim from seeing a health‐care provider of the opposite gender	
	Perpetrator imposing religious beliefs on victim and children	
	Perpetrator insisting on a dowry from victim or victim's family prior to or during marriage	
	Perpetrator forcing victim to work for pay	
2	Psychological Abuse	Moderate
	Perpetrator turning other people (e.g. children, family, friends) against victim	
	Perpetrator criticizing victim (e.g. bullying, belittling, demeaning, humiliating, ridiculing, constant put‐downs)	
	Perpetrator emotionally blackmailing victim (e.g. perpetrator threatening suicide or divorce)	
	Perpetrator making scenes that put down victim at social events	
	Perpetrator cursing and name calling victim	
	Perpetrator making victim feel they can never do anything right or are ever good enough	
	Perpetrator making victim feel that they are crazy	
	Perpetrator controlling and restricting family finances	
	Perpetrator accusing victim of having an affair	
	Perpetrator allowing external parties (e.g. business colleagues, extended family) to make or influence major family decisions (e.g. marriage, economic concerns) against victim's wishes	
	Perpetrator maintaining a secret lifestyle and/or withholding information about lifestyle from victim	
	Perpetrator manipulating and lying to victim	
	Perpetrator making hurtful comments about physical appearance of victim	
	Perpetrator publically denying any wrongdoing towards victim (e.g. in front of family, friends, etc.)	
	Perpetrator denying to the victim any wrongdoing within their relationship	
	Perpetrator frequently becoming jealous of victim	
	Perpetrator inappropriately blaming victim	
	Perpetrator sabotaging victim's housework (e.g. deliberately not eating home cooked meal)	
	Perpetrator ignoring victim	
3	Victim Response to Abuse	Low
	Victim destroying perpetrators personal property	
	Victim provoking perpetrator to use violence	
	Victim abusing perpetrator in response to abuse	
	Victim screaming and yelling at perpetrator	
	Victim criticizing perpetrator	
	Victim ignoring perpetrator	
4	Sexual Abuse	High
	Perpetrator infecting victim with sexually transmitted infections	
	Perpetrator injuring victim's breasts or genitals	
	Perpetrator forcing victim into sexual acts (e.g. unwanted: sodomy, viewing, pornography, oral sex, sex with animals)	
	Perpetrator punishing victim for not having sex	
	Perpetrator controlling sexual activity with victim (e.g. how often, contraception)	
	Perpetrator making unwanted sexually explicit comments to victim	
	Perpetrator keeping victim from enjoying sex	
5	Physical Abuse	**High**
	Perpetrator physically abusing victim (e.g. beating, slapping, pushing, spitting)	
	Perpetrator using a weapon to harm victim	
	Perpetrator using a weapon to intimidate or scare victim (e.g. knife, baseball bat)	
	Perpetrator forcing victim to consume alcohol and/or drugs	
	Perpetrator abusing victim as a result of alcohol and/or drug use	
	Perpetrator denying victim or children basic necessities (e.g. deny children clothing)	
	Perpetrator forcibly sleep depriving victim	
	Perpetrator demonstrating public displays of power over victim (e.g. silencing, grabbing victim in public)	
	Perpetrator abusing victim as a result of a gambling addiction	
	Perpetrator neglecting victim when they are sick	
6	Control	High
	Perpetrator keeping victim and children separated	
	Perpetrator controlling victim's important documents (e.g. passport, credit cards)	
	Perpetrator controlling victim's communications (e.g. emails and phone calls)	
	Perpetrator restricting or blocking victim's access to education or work	
	Perpetrator controlling victim's immigration activities (e.g. threatening deportation)	
	Perpetrating controlling and restricting daily activities of victim (e.g. when to do grocery shopping, haircut, banking)	
	Perpetrator controlling victim's social contact (e.g. victim cannot visit friends)	
7	Social and Emotional Manipulation	High
	Perpetrator interfering or blocking victim's access to health‐care providers	
	Perpetrator encouraging children to take part in violence towards victim (e.g. encouraging kids to act dismissive and demeaning towards the victim)	
	Perpetrator abusing victim as a result of victim's mental illness (e.g. depression, mood disorder)	
	Perpetrator treating victim like they are their own personal servant	
	Perpetrator abusing victim as a result of perpetrator's mental illness (e.g. depression, mood disorder)	
	Perpetrator encouraging family/friends to engage in abusive behaviours/language towards victim	
	Perpetrator publically humiliating victim	
	Perpetrator using aggressive behaviours intended to scare victim (e.g. punching a wall)	
	Perpetrator destroying victim's personal property	
	Perpetrator stalking victim	
	Perpetrator screaming and yelling at victim	
	Perpetrator controlling victim's physical appearance (e.g. victim told what to wear)	
	Perpetrator making victim cry	

Low = 3.42–3.84; Moderate = 3.854.27; High = 4.28–4.7 (bold is highest).

### The cluster map

Several regions of the cluster map were identified. First, there were two spatially distinct regions on the concept map, as illustrated by the division created by the bolded line in Fig. 1. The area on the right of the bolded line appears to be dominated by items that describe the actions and behaviours that characterize relationship stress and violence. A subdivision of this area may further be made, illustrated by the dotted line on Fig. 1, wherein the area below the dotted line is dominated by those actions or behaviours that represent a psychological component of relationship stress and violence. These include those actions or behaviours that fall into the domains of control, psychological abuse, and social and emotional manipulation. The close placement of these constructs suggests that participants perceived these categories to be conceptually similar. In contrast, the items that lie on top of the dotted line make up the actions or behaviours that represent sexual or physical components of relationship violence.

The relative distance between the items on the left of the bolded line (the influences and responses) and those on the right (the actions and behaviours) suggest that participants perceived them to be conceptually delineated. Those items composing the Victim's Response to Abuse cluster were particularly removed from the other items and may therefore be conceptually distinct from the actions and behaviours on the right of the bolded line. On the other hand, items in the External and Cultural Influences cluster, which speak to the circumstances and factors that affect the development and continuation of relationship violence, appear to be conceptually close to the items describing actions and behaviours of relationship stress and violence based on their proximity on the map. For example, item 38 (making sexist and racist remarks) in the External and Cultural Influences cluster is conceptually similar to item 68 (slanting cultural, religious and moral values) in the Psychological Abuse cluster.

Clusters with low bridging values also indicate greater cohesion within the cluster as those items are more often sorted together by participants. Almost all clusters had relatively low bridging values, especially ‘Psychological Abuse’ and ‘Social and Emotional Manipulation’ (see Table 2). At the other end of the spectrum, ‘Victim's Response to Abuse’ (bridging value 0.84) and ‘Sexual Abuse’ (bridging value 0.41) had higher values meaning that participants often sorted items within that cluster with items in other areas of the map, suggesting that the cluster may not be internally cohesive (see Tables 1 and 2).

**Table 2:** Cluster average bridging values

No.	Clusters	Average bridging values
1	External and Cultural Influences	0.30
2	Psychological Abuse	0.19
3	Victim Response to Abuse	0.84
4	Sexual Abuse	0.41
5	Physical Abuse	0.26
6	Control	0.25
7	Social and Emotional Manipulation	0.21

### Rating results

Participants rated each item in terms of its centrality to their definition of partner abuse and these item ratings were averaged over each cluster. The average ratings across participants for all items fell in the range of 3.28–4.87, meaning that participants tended to rate items in the upper range of the 5‐point scale. Item 81: ‘Perpetrator physically abusing victim (e.g. beating, slapping, pushing, spitting)’ received the highest average rating on the Defining Relationship Violence Scale. Item 16: ‘Victim ignoring the perpetrator’, received the lowest. The average cluster ratings fell between 3.71 and 4.53. On average, items in the Physical Abuse cluster were rated as most central to the definition of relationship violence, followed by items in the Sexual Abuse cluster and then the Control cluster; while items in the Victim Response to Abuse cluster were, on average, rated lower.

The correlation or level of agreement between the average cluster ratings for subgroups (e.g. comparing those with high versus low income), ranged from *r‐*values of 0.94–1.0 (i.e. almost perfect or perfect agreement), suggesting that the conceptualization of partner violence did not differ by any of the sociodemographic or attitudinal variables (see Table 3).

**Table 3:** Pearson's correlation coefficients (*r*‐values) for ‘Definition of Relationship Violence’ ratings by subgroups of sociodemographic and attitudinal variables

Sociodemographic or attitudinal variable	Subgroup (*n*)^a^	Subgroup (*n*)^a^	*R‐*value
Education	Has not received a post‐secondary degree (13)	Received Post‐secondary Degree(s) (45)	0.94
Relationship status at time of study	In a Relationship (29)	Not in a Relationship (32)	0.98
Marital status^b^	Single (never married) OR Widowed (23)	Married OR Common Law (29)	0.98
	Married OR Common‐Law (29)	Separated OR Divorced (9)	1.0
Decision Making (DM) Scale (median = 1.2, *out of 5*)	Scored <1.2 (29)	Scored >1.2 (24)	0.94
DM1 wife work	(Strongly) Disagree (48)	Neutral OR (Strongly) Agree (13)	0.94
DM2 wife go out	(Strongly) Disagree (47)	Neutral OR (Strongly) Agree (14)	0.97
DM3 man head of house	(Strongly) Disagree (40)	Neutral OR (Strongly) Agree (21)	0.99
DM4 man force sex	Strongly Disagree (46)	Disagree OR Neutral (14)	0.97
DM5 man decide money	Strongly Disagree (40)	Disagree, Neutral OR (Strongly) Agree (21)	0.96
Annual household incomeA	$50 K and below (33)	Above $50 K (38)	0.98
Age^b^	<30 years (13)	31–44 years (48)	0.96
	31–44 years (12)	Over 45 years (48)	0.99

a Items do not total to the full sample due to missing data on the surveys.

b Because there are three marital status categories, single, married and separated/divorced, we performed two subgroup correlation comparisons for this variable. Also, there were 3 age categories, >30 years, 31–44 years and >45, and we performed two subgroup correlations for this variable.

### Comparison to existing measures of IPV

The cluster domains obtained from concept mapping were compared to the domains of eight commonly used instruments for measuring IPV in research and screening (see Table 4). We found that none of the instruments covered all of the domains generated through the concept mapping process. However, taken together, all but one of the clusters (the External and Cultural Influences cluster) was represented by more than one of the published instruments and all but two (External and Cultural Influences cluster and Victim Response to Abuse) represented in the instruments most recently developed.^17^, ^35^

**Table 4:** Frequently used intimate partner violence scales compared to domains from concept mapping

	Physical Abuse	Psychological Abuse	Sexual Abuse	Control	Victim Response to Abuse	External and Cultural Influences	Social and Emotional Manipulation	Other concepts not in the domains
Concept Mapping Domains from current study	X	X	X	X	X	X	X	
Abuse Assessment Screen (AAS)^40^	X	X	X					Fear of partner, injury, context of pregnancy
Revised Conflict Tactics Scales (CTS2) and Short Form^37^, ^41^	X	X	X		X			Negotiation
Hurt, Insulted, Threatened with harm and Screamed at them (HITS)^42^	X	X					X	
Women's Experience with Battering (WEB)^36^	X	X		X	X			Entrapment, Disempowerment
Checklist of Controlling Behaviors (CCB)^17^	X	X	X	X			X	
Composite Abuse Scale (CAS)^35^	X	X	X	X			X	Harassment
Woman Abuse Screening Tool (WAST)^43^	X	X	X					

External and Cultural Influences, Social and Emotional Manipulation and Victim's Response to Abuse were the three clusters least represented in the published instruments. With the exception of the Women's Experiences of Battering (WEB), none represented the domain of the Victim's Response to Abuse cluster, although the CTSR considers the possibility that the actions described in the instrument are bidirectional. Three instruments, the HITS, Checklist of Controlling Behaviors (CCB) and Composite Abuse Scale (CAS), represented components of Social and Emotional Manipulation.

There are differences in the way that domains were conceptualized in concept mapping compared with the published instruments assessed. That is, the composition of items within domains that were similar across both the cluster map and the instruments were not the same. In some instances it was clear that differences arose as a result of the intent behind the measurement of the scale items. For example, unlike the items in the concept mapping study that describes events/occurrences that characterize relationship violence, the items in the WEB describe the cognitive experiences of women who are victims of relationship violence.^36^ Similarly, items composing the Sexual Coercion subscale of the CTSR are similar in nature to the sexual abuse cluster, but are different in that items in the CTSR are restricted to the assessment of the presence of coercive acts ranging from verbal to physical, which are intended to compel the partner to engage in unwanted sexual activity.^37^

## Discussion

In the last three decades, dozens of instruments have emerged measuring partner violence. Instruments that have emerged more recently (e.g. CAS and CCB) have been responsive to the growing literature that calls for an expansion of the conceptualization of IPV.^17^, ^35^ We elicited ideas about what comprises ‘severe relationship conflict, abuse, violence and neglect’ from a diverse sample of men and women. Asking both men and women was an important advancement in the field as too few studies ask men about their perceptions of relationship conflict and partner violence.^19^ Concept mapping was an ideal tool for our research question as we were able to elicit and preserve participants’ ideas and statements about relationship conflict and abuse.^32^ Unique strengths of concept mapping include data gathering activities that facilitate equal representation of all individuals’ views during sorting and rating, while also incorporating group consensus during the mapping process. Statistical treatment of the data adds rigour to the qualitatively generated ideas and heavy participant involvement in brainstorming and mapping ensures that the final results represent the ideas and words of the target population.

Participants in our sample identified concepts and domains that go beyond what is typically included in existing measures. One domain, rated as moderately important in the conceptualization of IPV by our participants, External and Cultural Influences, included items such as use of cultural values to excuse abusive or violent behaviour and blaming victims for the (non‐male) gender of a child. These items likely reflected what our South Asian participants experienced as relationship conflict and abuse and illustrate how existing measures have failed to capture diverse abusive experiences. Another domain rarely represented in existing measurement tools was Victim Response to Abuse. These items represented selected victim's provocation and behaviours towards perpetrators and this domain was rated as having low importance in the conceptualization of partner violence. This construct deserves examination in future research to ensure that this construct does not serve to ‘blame the victim’ if included in future instruments. Social and Emotional Manipulation was a domain rated of high importance by our participants and is rarely included in existing measures of IPV. Example items include public humiliation of victims; aggressive behaviours intended to scare the victim; encouraging children or family/friends to engage in abusive behaviours towards the victim; and abuse resulting from the victim's or the perpetrator's mental illness. In some measures, some of these items might appear in psychological abuse domains.^6^ Yet, participants rated this domain as being highly important to the idea of partner violence, suggesting that it is an area to investigate further when developing tools to measure violence in the future. Other domains rated ‘high’ by our participants in terms of centrality to the notion of IPV include Control, Physical Abuse and Sexual Abuse. Psychological Abuse was rated as moderately important as a concept of IPV by our sample.

We found uniform consistency across subgroups of age, income and relationship attitudes in terms of how the items were rated for centrality to the idea of partner violence. We had expected to see differences by income because socio‐economic position is such a strong predictor of the occurrence of IPV and that this association can differ by type of IPV,^13^, ^38^ but no significant differences were observed as correlations were all close to 1.0. Our sample sizes were also adequate to demonstrate differences in these correlations if they had existed.

The limitations of our work include the use of a convenience sample. However, it was our goal to ensure that we had a diverse population and that we did not have an overrepresentation of ‘White women who have some available personal resources’.^10^ Our sample of men was smaller than we had intended as they were harder to recruit and less eager than women to discuss this topic. We had also hoped to include a larger sample of Aboriginal women and men. Our sample included more than one individual who identified as Aboriginal as we held a group brainstorming session at an Aboriginal community organization, but several participants did not identify themselves as such on our demographic survey. An Aboriginal‐specific, community‐partnered participatory action research approach may facilitate future efforts to better understand and address.^39^

We only examined forms of abuse in this study and did not seek to gain perspectives on how this sample perceived severity of abuse or relationship types (e.g. adolescent relationships, dating violence or elder abuse). While the literature identifies the need to better understand the context of abuse, we were only able to examine context as it was mentioned in our items. Thus, family involvement or abusive behaviours in public were mentioned, but this was not explored in depth. Moreover, we sought to examine the influence of being a victim of abuse on perceptions of the centrality of behaviours and attitudes that comprise partner violence. However, many participants refused to answer those questions and after removing the missing data, the final sample was too small to analyse. Additionally, missing information about demographic and attitudinal data meant that not all participants could be included in those analyses.

The composition of our sample, that is the high proportion of participants with post‐secondary education and with East and South Asian background, may have driven the findings in terms of content and importance. However, the sample did contain diversity with regard to demographics and attitudinal factors. Moreover, we found no differences by subgroups of these attitudinal and demographic factors, suggesting that our findings can be generalized across these characteristics. In this analysis, we were seeking to assess the centrality of items across the full sample. However, given the reports in the literature of how cultural and contextual factors may influence group‐specific notions of IPV, future work might explore subpopulation‐specific conceptualizations and ratings of domains.

The fact that many of the items from the Social and Emotional Manipulation and Social and Cultural Influences are left out of almost all commonly used and existing measures^10^ has implications for the measurement of the prevalence and types of partner violence. Currently, measures used in large population typically focus on a range of physical abuse experiences with little or no emphasis on sexual abuse or verbal abuse. Nevertheless, our findings suggest that some existing instruments may be asking about the relevant experiences that men and women from diverse backgrounds perceive as partner violence and never capture the extent to which victims experience abuse due to social or cultural influences or emotional manipulation among others.

This problem of underestimation in the existing literature should be remedied by improving and expanding the range of IPV experiences included in population‐based surveys. A further consequence of failing to acknowledge or measure the social and cultural influences on IPV can lead to improperly designed prevention and intervention programmes. Thus, our findings support the growing literature that current measures of IPV should be modified to include the experiences of diverse populations for accurate measurement and program design.
